# Ghrelin Attenuates Neuroinflammation and Demyelination in Experimental Autoimmune Encephalomyelitis Involving NLRP3 Inflammasome Signaling Pathway and Pyroptosis

**DOI:** 10.3389/fphar.2019.01320

**Published:** 2019-11-06

**Authors:** Fei Liu, Zijian Li, Xin He, Haiyang Yu, Juan Feng

**Affiliations:** Department of Neurology, Shengjing Hospital of China Medical University, Shenyang, China

**Keywords:** ghrelin, multiple sclerosis, experimental autoimmune encephalomyelitis, inflammasome, pyroptosis

## Abstract

Multiple sclerosis (MS) is a chronic autoimmune and degenerative disease of the central nervous system, and conventional treatments have limited efficacy or side effects. Ghrelin, a 28-amino acid octanoylated peptide, has been reported to have neuroprotective effects, including anti-oxidation, anti-inflammation, and anti-apoptosis. Pyroptosis, also called inflammatory cell death, is triggered by overly active inflammasomes and accompanied by the production of numerous cytokines. As immune dysfunction is primarily involved in the pathogenesis of MS, this study aimed to explore the therapeutic effects and precise functional mechanisms of ghrelin against the nod-like receptor protein 3 (NLRP3) inflammasome and pyroptosis in experimental autoimmune encephalomyelitis (EAE). Sprague Dawley rats were immunized with guinea pig spinal cord homogenates and pertussis toxin to develop an EAE model. All rats were randomly divided into four groups: normal control group, EAE group, EAE + ghrelin group, and ghrelin control group. EAE rats showed abnormal behavioral scores and body weight changes. Histologic analysis displayed severe inflammatory infiltration and demyelination in the brain and spinal cord of EAE rats. Ghrelin treatments potently restored these abnormal changes. In addition, the ghrelin-treated EAE group showed significantly downregulated expression of inflammatory cytokines. The expression of proteins involved in the NLRP3 signaling pathway and pyroptosis was decreased as well. We also found that the anti-inflammatory effect of ghrelin was associated with inhibition of nuclear factor (NF)-κB activation. Compared with rats in the healthy control group, rats in the ghrelin control group did not show statistically significant changes in histologic examinations, pro-inflammatory cytokines production, or molecules involved in the NLRP3 signaling pathway, which indicated that ghrelin induced no side effects in the animals of our study. Our findings provide more insight into the use of ghrelin as a novel candidate for MS.

## Introduction

Multiple sclerosis (MS) is a chronic autoimmune and degenerative disease of the central nervous system (CNS) that mainly occurs in young adults, with a female predominance (Lassmann, 1999). The characteristic pathologic changes in MS patients are multifaceted, including multiple demyelinating plaques in the white matter of the CNS, predominantly located around the lateral ventricles and accompanied with reactive glial hyperplasia and axonal injury (Correale et al., 2017). Despite increased research into the etiology, pathogenesis, and treatment of MS, current therapeutic regimes for MS are relatively limited, and the disease continues to be a therapeutic challenge worldwide.

Although the etiology and mechanism of MS remain elusive, neuroinflammation was shown to play a pivotal role in the occurrence and development of MS (Barclay and Shinohara, 2017). Excessive inflammation leads to cell death and tissue damage, such as demyelinating lesions and axonal injuries. The nod-like receptor protein 3 (NLRP3) inflammasome, a promoter involved in innate immune response initiation, has been reported to participate in the progression of many neurodegenerative diseases, including MS (Inoue and Shinohara, 2013; Song et al., 2017; Wang et al., 2019). Pyroptosis is a newly discovered form of inflammatory cell death that can be triggered by activation of the NLRP3 inflammasome (Shi et al., 2017a). Once the NLRP3 inflammasome is assembled, functional caspase-1 is released, thus activating interleukin (IL)-1β, IL-18, and gasdermin D (GSDMD), an important downstream target protein in pyroptosis (He et al., 2015; Semino et al., 2018). As a result, cells swell until their membranes burst, releasing a large number of inflammatory cytokines and leading to a dangerous inflammatory cascade (Chen et al., 2016). Therefore, blocking the NLRP3 signaling pathway and pyroptosis is expected to alleviate neuroinflammation and disease progression.

Ghrelin, a 28-amino acid octanoylated peptide first extracted from rat stomachs in 1999, acts as an endogenous ligand for the growth hormone secretagogue receptor (GHSR) (Kojima et al., 1999). GHSR is expressed in many tissues, indicating that ghrelin has various biologic functions. During starvation, ghrelin is produced chiefly in gastric mucosal endocrine cells and also secreted in the arcuate nucleus of the hypothalamus (Goldstein et al., 2011). Ghrelin has several endocrine activities, including the regulation of growth hormone secretion, appetite, dietary cravings, and body composition (Scerif et al., 2011; Steyn et al., 2016). Accumulating evidence suggests that ghrelin can pass through the blood–brain barrier (BBB) and exert non-metabolic functions as a neuropeptide in the CNS (Rhea et al., 2018). For instance, ghrelin has a protective role in MS with favorable effects that are related to neuroinflammation attenuation associated with Th1/Th17-driven immune responses and regulatory T-cell production (Souza-Moreira et al., 2013). However, whether ghrelin participates in the modulation of inflammasome-related neuroimmunity needs further research.

In this study, we examined the anti-demyelinating and anti-neuroinflammatory effects of ghrelin to study its effects in an experimental autoimmune encephalomyelitis (EAE) rat model, an ideal MS animal model (Baker and Amor, 2014). We also studied the molecular changes involving the NLRP3 inflammasome pathway before and after ghrelin administration and explored the effects of ghrelin on the pyroptosis signaling pathway. This study provides novel information on the effects of ghrelin as a neuroprotectant and as a possible therapy for MS patients.

## Materials and Methods

### Drugs and Reagents

Ghrelin was obtained from Enzo Life Sciences (New York, USA) and dissolved in PBS. A bicinchoninic acid (BCA) protein assay kit was obtained from Beyotime Biotechnology (Shanghai, China). Nitrous oxide (NO) and lactate dehydrogenase (LDH) assay kits were purchased from the Nanjing Jiancheng Institute of Biological Engineering (Nanjing, China). Anti-NLRP3 and anti-apoptosis-associated speck-like protein containing a CARD (ASC) antibodies were obtained from Novus Biologicals (Centennial, USA). Anti-caspase-1, anti-CD68, and anti-inducible nitric oxide synthase (iNOS) antibodies were obtained from Abcam (Cambridge, UK), anti-IL-1β, anti-NF-κB P65, and anti-NF-κB p-P65 antibodies were obtained from Cell Signaling Technology (Danvers, USA). An anti-GSDMD antibody was obtained from Santa Cruz Biotechnology (Texas, USA). Anti-iba1 and anti-β-actin antibodies were obtained from Proteintech (Chicago, IL, USA). The luxol fast blue (LFB) stain fluid was obtained from Sigma-Aldrich (St. Louis, MO, USA). The enzyme-linked immunosorbent assay (ELISA) kits for rat IL-1β, IL-6, and tumor necrosis factor-α (TNF-α) were purchased from Dakewe Biotech (Shenzhen, China). The ELISA kit for rat IL-18 was purchased from RayBiotech (Norcross, USA).

### Animals

Female Sprague Dawley (SD) rats (200–220 g) and guinea pigs (350–450 g) were procured from Beijing HFK Bioscience Corporation, China. Animals were housed with free access to food and water under a 12 h day/night cycle in a specific-pathogen-free level animal laboratory. Standard room environment was maintained with a constant temperature of 23 ± 3°C and relative humidity of 55 ± 3%. Experiments were strictly performed in accordance with the NIH guidelines for the Care and Use of Laboratory Animals. All experimental procedures on animals in this work were approved by the Institutional Animal Care and Use Committee of ShengJing Hospital, China Medical University (no. 2016PS012K). The number of animals used and animal suffering were minimized as much as possible.

### Development of the EAE Model

The EAE model was established as previously described (DellaValle et al., 2016). Briefly, all animals were initially acclimatized to the environment. Complete Freund’s adjuvant (CFA) was prepared using incomplete Freund’s adjuvant (Sigma, St. Louis, MO, USA) and 10 mg/ml *Mycobacterium tuberculosis* H37Ra (Difco, BD Biosciences, USA). Guinea pig spinal cord homogenates (1 g spinal cord mixed with 1 ml 0.9% saline) were added to the same volume of CFA and thoroughly emulsified. On days 0 and 7, each rat was immunized with the emulsion by subcutaneous injection into both hind footpads and the base of the tail with a total volume of 0.4 ml. Pertussis toxin (PTX, Sigma, St. Louis, MO, USA) was injected subcutaneously into rats at days 0 and 2.

### Experimental Grouping

Animals were randomly grouped as follows: Group 1, healthy control (PBS injection; *n* = 10); Group 2, EAE (*n* = 10); Group 3, EAE + ghrelin (100 µg/kg, once daily; *n* = 10); and Group 4, ghrelin control (100 µg/kg, once daily; *n* = 10). PBS and ghrelin were injected subcutaneously. The dose of ghrelin used in our experiments was consistent with that used in similar studies (Chang et al., 2019; Ling et al., 2019). According to our previous work, the peak EAE onset, on about day 14, was a suitable time to collect blood samples and acquire brain and spinal cord (lumbar enlargement) tissues (Yang et al., 2016).

### Behavioral Assessments

Clinical behavioral scores of experimental animals in each group were blindly recorded by two observers each day according to the following criteria: 0, no clinical symptoms; 1, tail tension disappeared or slightly clumsy gait; 2, flaccid hind limb; 3, moderate hind limb paralysis; 4, paralysis of both hind limbs, paralysis of the forelimbs, or weakened muscle strength with urinary and fecal disorders; and 5, pre-death stage; ± 0.5 units were placed between each criterion. Changes in animal weights were also examined daily.

### Hematoxylin and Eosin (H&E) and LFB Staining

After anesthesia, the hearts of the rats were perfused with normal saline and then 4% paraformaldehyde. After humane euthanasia, the brain and spinal cord tissues were separated and fixed in 4% paraformaldehyde for 24 h, then dehydrated with graded ethanol, and transparentized with xylene. After being embedded in paraffin, the tissues were sectioned into 5-µm-thick slices for H&E or LFB staining to assess the degree of inflammatory cell infiltration and spinal demyelination, respectively, following the manufacturers’ protocols. Inflammatory infiltrations and demyelination were evaluated as described in a previous study, and the final score of each rat was averaged from three different histologic sections (Qiu et al., 2018). Stained sections were observed under a Nikon 300 microscope.

### Immunohistochemistry (IHC)

Protocols were obtained from our previous experiments (He et al., 2017). Five-micrometer-thick paraffin sections were treated with 3% H_2_O_2_ and goat serum albumin for endogenous peroxidase inactivation and nonspecific binding site blocking. Next, sections were incubated with anti-CD68 and anti-iba1 primary antibodies overnight at 4°C and biotin-labeled goat anti-rabbit or mouse IgG for 30 min at 37°C in sequence. Finally, diaminobenzidine (DAB) as the chromogen was used to visualize the immunocomplexes that were captured under a Nikon 300 microscope.

### Real-Time Polymerase Chain Reaction (RT-PCR) Analyses

We used a two-step quantitative RT-PCR for our experiments. Total RNA was isolated from spinal cord tissues using RNAiso Plus (TaKaRa, Japan) according to the manufacturer’s instructions, and then RNA concentration was quantified. A certain amount of RNA was reverse-transcribed into single-stranded cDNA using the TaKaRa PrimeScript^™^ RT Master Mix. Then, cDNA samples were used to quantify gene expression levels using the TaKaRa TB Green^™^ Premix Ex Taq^™^-based PCR. The mRNA expression level of each sample was calculated using the 2-ΔΔCt method and was normalized with an endogenous reference, GAPDH. Primer sequences used in this study are listed in **Table 1**.

**Table 1:** Primer sequences used for RT-PCR.

Gene	Sequences (5′ to 3′)	
TNF-α	Forward	TGAACTTCGGGGTGATCG
	Reverse	GGGCTTGTCACTCGAGTTT
IL-6	Forward	AGAAAAGAGTTGTGCAATGGCA
	Reverse	GGCAAATTTCCTGGTTATATCC
iNOS	Forward	CACCACCCTCCTTGTTCAAC
	Reverse	CAATCCACAACTCGCTCCAA
COX-2	Forward	GCAAATCCTTGCTGTTCCAACC
	Reverse	GGAGAAGGCTTCCCAGCTTTTG
IL-1β	Forward	TGGCAGCTACCTATGTCTTGC
	Reverse	CCACTTGTTGGCTTATGTTCTG
IL-18	Forward	AAACCCGCCTGTGTTCGA
	Reverse	TCAGTCTGGTCTGGGATTCGT
GAPDH	Forward	GACATGCCGCCTGGAGAAAC
	Reverse	AGCCCAGGATGCCCTTTAGT

### Western Blotting Analyses

Lysis buffer with added protease and phosphatase inhibitors was used to extract protein from minced spinal cord tissues. After the tissues were incubated on ice and centrifuged, supernatants were collected, and the BCA protein assay kit was used to measure protein concentrations. Then, the supernatant was mixed with an SDS-PAGE sample loading buffer and heated in a mental bath. Normalized samples (35 µg) were loaded to SDS-PAGE electrophoresis and transferred to PVDF membranes (Millipore). After being blocked in 5% skimmed milk for 2 h at room temperature, membranes were then incubated overnight at 4°C with primary antibodies against NLRP3 (1:500), caspase-1 (1:1,000), ASC (1:1,000), IL-1β (1:1,000), GSDMD (1:500), NF-κB p65 (1:1,000), NF-κB p-P65(1:1,000), iNOS (1:1,000), or β-actin (1:2,500). Next, the membranes were washed with a TBST buffer (Tris-buffered saline containing 0.1% Tween 20, pH = 7.6) and secondary antibodies for 2 h at room temperature on a shaking table. A chemiluminescence imager was used to detect chemiluminescence membrane immunoreactivity using the chemiluminescence detection kit (ECL kit, Millipore). Relative immunoreactivity levels were reflected using grayscale values and were standardized by a reference protein (β-actin) using ImageJ software (NIH).

### ELISA

Sample preparation was carried out firstly. All the test specimens were disposed and analyzed following the manufacturer’s protocols. The ELISA kits for rat IL-1β, IL-18, IL-6, and TNF-α were used to detect the expressions of these inflammatory cytokines in serum collected from rats. The results were compared to standard curve with gradient concentration. Absorbance (Optical density value) was measured at the appropriate wavelength recommended by protocols. All experiments were repeated three times.

### Nitrite Analyses

In this study, nitrite generated from rat serum was detected to estimate the expression of NO indirectly. All the test specimens were processed and analyzed in accordance with manufacturer’s procedures. Nitrite absorbance (optical density value) was measured at 550 nm wavelength by a microplate reader.

### LDH Analyses

Blood was first collected from rats and then centrifuged to obtain serum. Using the microwell plate method, serum LDH concentrations were measured using a commercial LDH assay according to the manufacturer’s protocols. The colorimetric wavelength was determined at 450 nm. All experiments were carried out in triplicate.

### Statistical Analysis

Data were expressed as the mean ± SD from at least three independent experiments. Statistical analysis of weight changes and clinical behavioral scores were performed using two-way analyses of variance (ANOVA) followed by Bonferroni’s multiple group comparisons. Other results were performed with one-way ANOVA. *P* values < 0.05 were considered statistically significant.

## Results

### Ghrelin Delays the Onset and Relieves the Symptoms of EAE Rats

**Figure 1:**
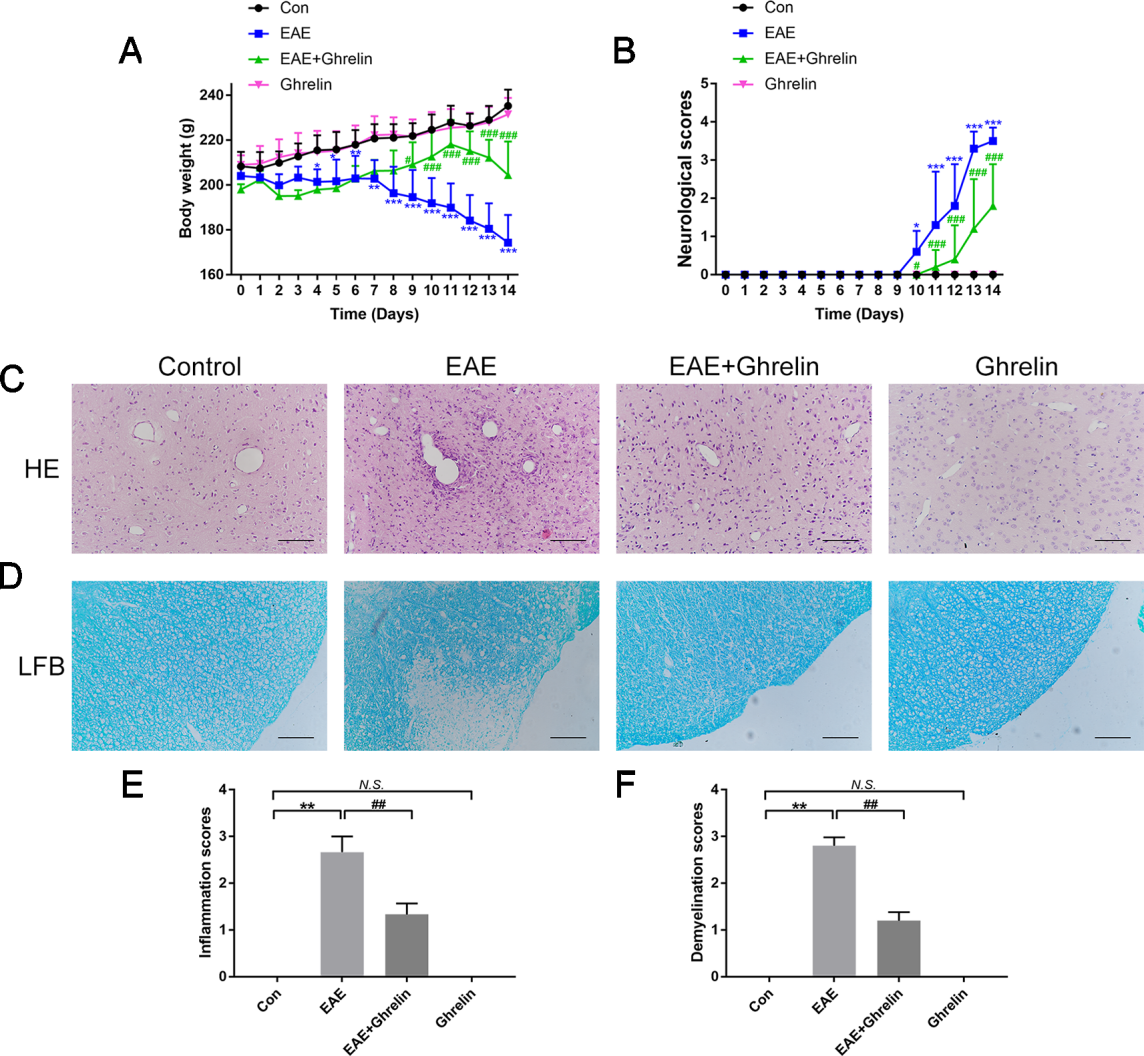
Treatment of ghrelin ameliorated symptoms and suppressed inflammatory infiltration and demyelination in CNS tissues of EAE rats. **(A)** Body weight changes of rats during the experimental period (*n* = 10). **(B)** Variations of neurological scores during the experimental period (*n* = 10). Statistical analysis of different groups was performed with two-way ANOVA followed by Bonferroni’s multiple group comparison. **(C)** H&E staining showed that ghrelin attenuated inflammatory infiltration in brain tissues of EAE rats. **(D)** LFB staining showed that ghrelin attenuated demyelination in spinal cord tissues of EAE rats. Inflammation scores (*n* = 5) **(E)** and demyelination scores (*n* = 5) **(F)** were markedly decreased in the ghrelin-treated group. Scale bars: 50 µm. Data were shown as mean ± SD. *P 0.05, **P 0.01,***P 0.001 versus the control group, ^#^ *P* 0.05, ^##^ *P* 0.01, ^###^ *P* 0.001 versus the EAE group. *NS*, not significant. Con, control.

In the present study, the EAE rat model of MS was successfully established, which laid the foundation for the following experiments. Weight changes (**Figure 1A**) and clinical behavioral scores (**Figure 1B**) were recorded every day to evaluate the therapeutic effects of ghrelin on MS in EAE rats. EAE rat body weights decreased at day 8 and declined at a faster rate during the following days, which was consistent with our previous studies (Li et al., 2019). Clinical behavioral line chart scores indicated that EAE group animal conditions worsened at day 10 compared with the healthy control group. After ghrelin treatment, EAE rat body weights began to decrease at day 12, which was later than untreated EAE rats. In addition, ghrelin-treated EAE rats appeared to have an uncoordinated performance even later, and daily mean neurologic scores were lower compared with those of the untreated EAE rats. These results demonstrated that ghrelin treatments not only delayed the onset of EAE but also alleviated the symptoms of EAE rats.

### Ghrelin Reduces Inflammatory Brain Infiltrations and Spinal Cord Demyelination in EAE Rats

The rats were sacrificed to obtain CNS tissues at the peak onset of disease, which was about day 14 post-inoculation. We performed H&E staining on brain tissues to evaluate inflammatory cell infiltration (**Figures 1C, E**). EAE rats suffered from more serious inflammation, as shown by apparent perivascular cuffs and diffuse inflammatory cell infiltrations around small vessels. Ghrelin treatment markedly ameliorated inflammatory cerebral cell infiltrations. Moreover, when we stained lumbar spinal cord enlargements with LFB to assess the protective effects of ghrelin on demyelination (**Figures 1D, F**), we found a statistical decline in demyelination scores in ghrelin-treated EAE rats compared with those that did not receive treatment. Therefore, these results demonstrated that ghrelin decreased the severity of EAE by controlling CNS inflammation and demyelination.

### Ghrelin Decreases Inflammatory Cytokines Release in EAE Rats

**Figure 2:**
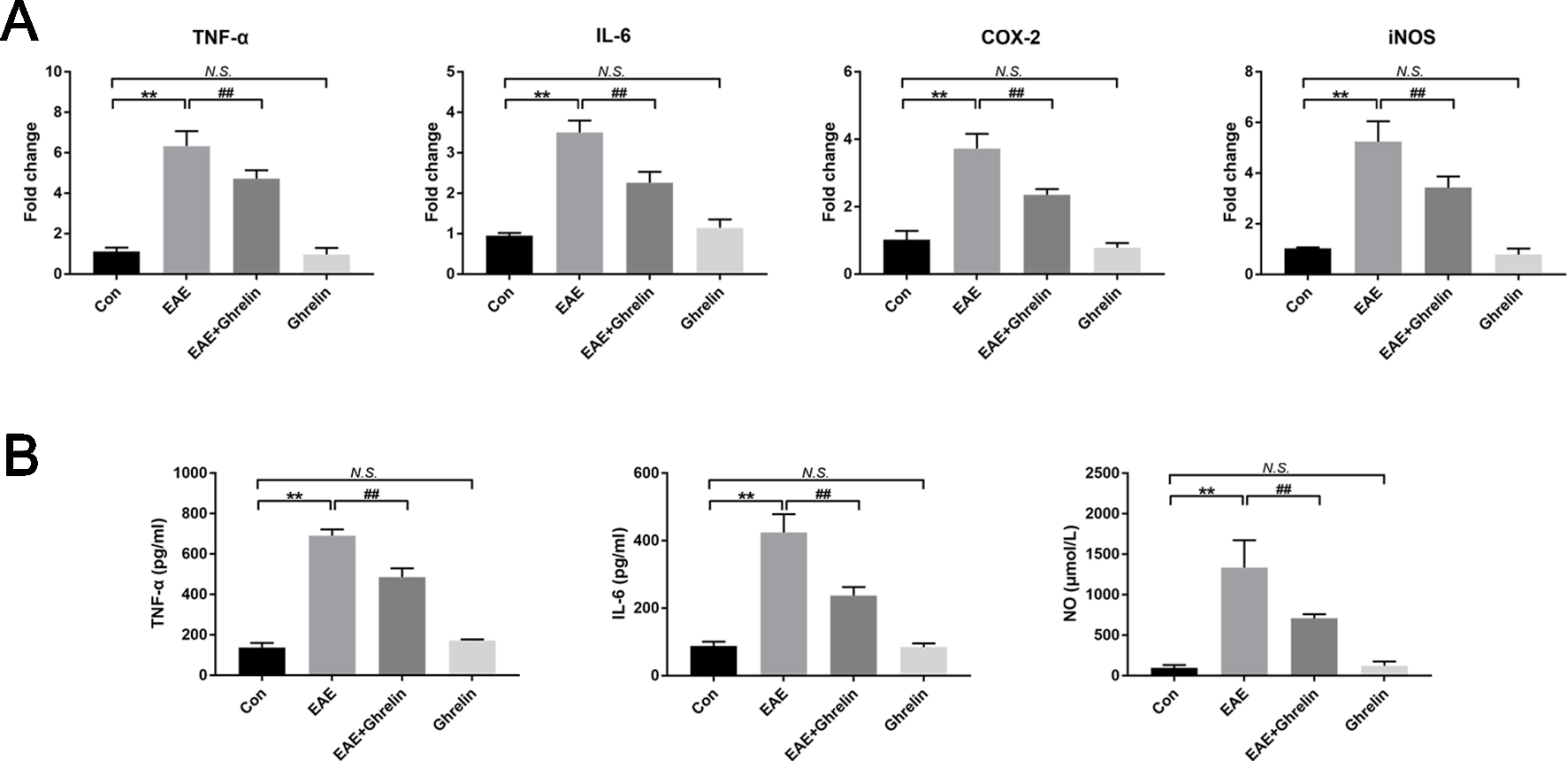
Treatment of ghrelin decreased the production of inflammatory cytokines of EAE rats. **(A)** The mRNA expressions of inflammatory cytokines were measured by quantitative RT-PCR (*n* = 5). **(B)** Inflammatory cytokine expressions in serum were measured by ELISA (*n* = 10). Expression of NO in serum was measured by a NO assay kit (*n* = 10). Data were shown as mean ± SD.**P 0.01 versus the control group, ^##^ *P* 0.01 versus the EAE group. *NS*, not significant. Con, control.

To reaffirm anti-inflammatory effects of ghrelin following the EAE challenge, we further assessed the expression of some pro-inflammatory cytokines produced by tissue-invading immune cells. Results of RT-PCR revealed that TNF-α, IL-6, COX-2, and iNOS mRNA were markedly expressed in the spinal cord of EAE rats compared with levels in healthy control rats (**Figure 2A**). Marked increases in TNF-α, IL-6, and NO expressions were also observed in serum of EAE rats, which was consistent with RT-PCR results (**Figures 2B, C**). Nevertheless, ghrelin treatments dramatically reduced these changes, which convincingly demonstrated the anti-inflammatory effects of ghrelin.

### Ghrelin Suppresses Microglia Aggregation and Activation in EAE Rats

**Figure 3:**
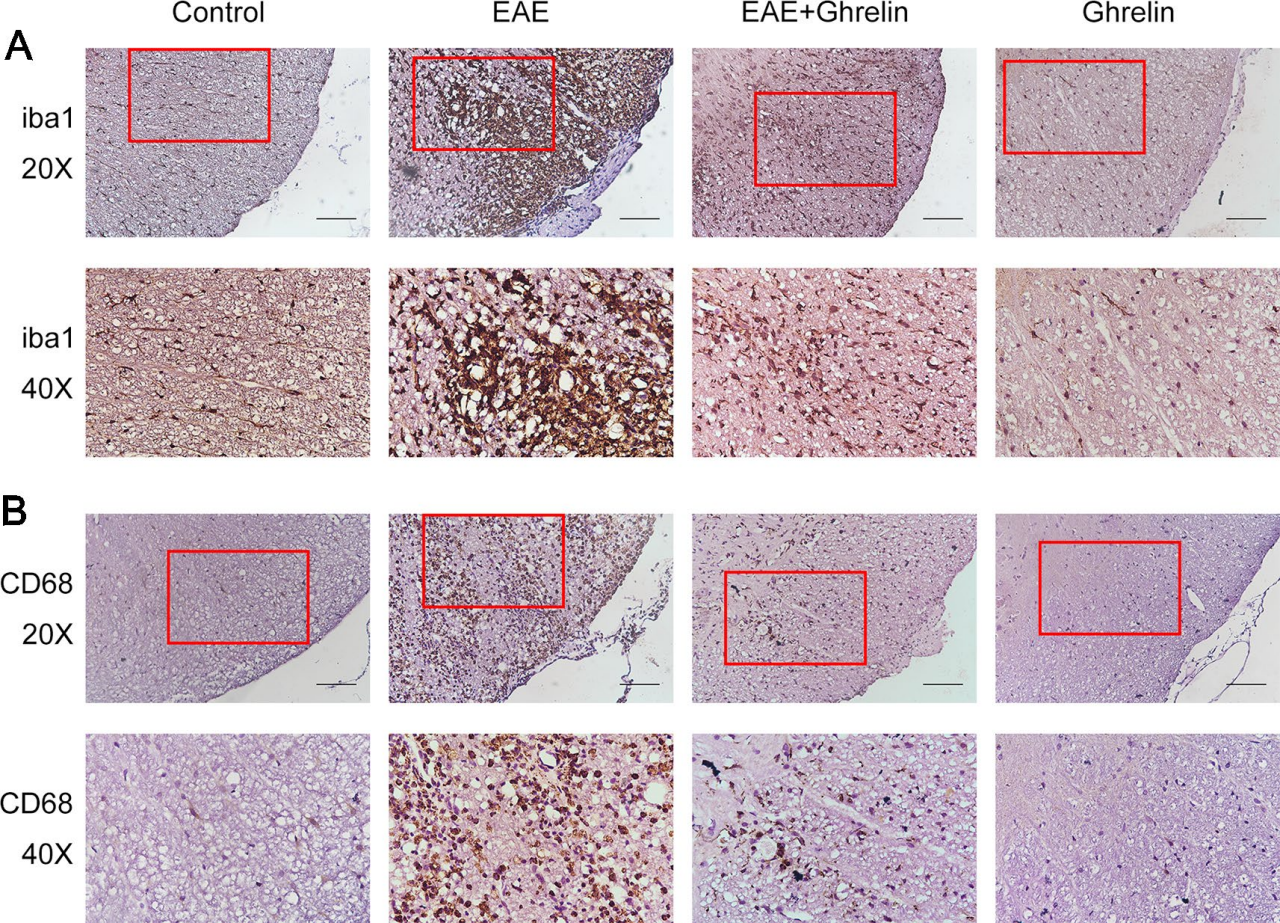
Treatment of ghrelin inhibited microglia aggregation and activation in EAE rats. IHC staining of iba1 **(A)** and CD68 **(B)** in the spinal cord tissues. Scale bars: 50 µm.

Iba1, a calcium-binding protein specific to microglia, was detected by IHC to exhibit microglia aggregation. Compared with the healthy control group, the EAE group showed that more microglia concentrated around the damaged tissues and small vessels (**Figure 3A**). CD68, a lysosomal protein with high expression on the surface of activated microglia versus low expression on resting microglia, was measured in lumbar enlargements of the spinal cord (**Figure 3B**). CD68 expression was upregulated in EAE rats compared with the healthy control group rats, which represented that more microglia were activated after EAE. Interestingly, ghrelin treatments decreased positive rates of both iba1 and CD68 expressions as shown in **Figures 3A, B**, which indicated that ghrelin could suppress microglia aggregation and activation.

### Ghrelin Decreases the NLRP3 Signaling Pathway Molecular Expression in EAE Rats

**Figure 4:**
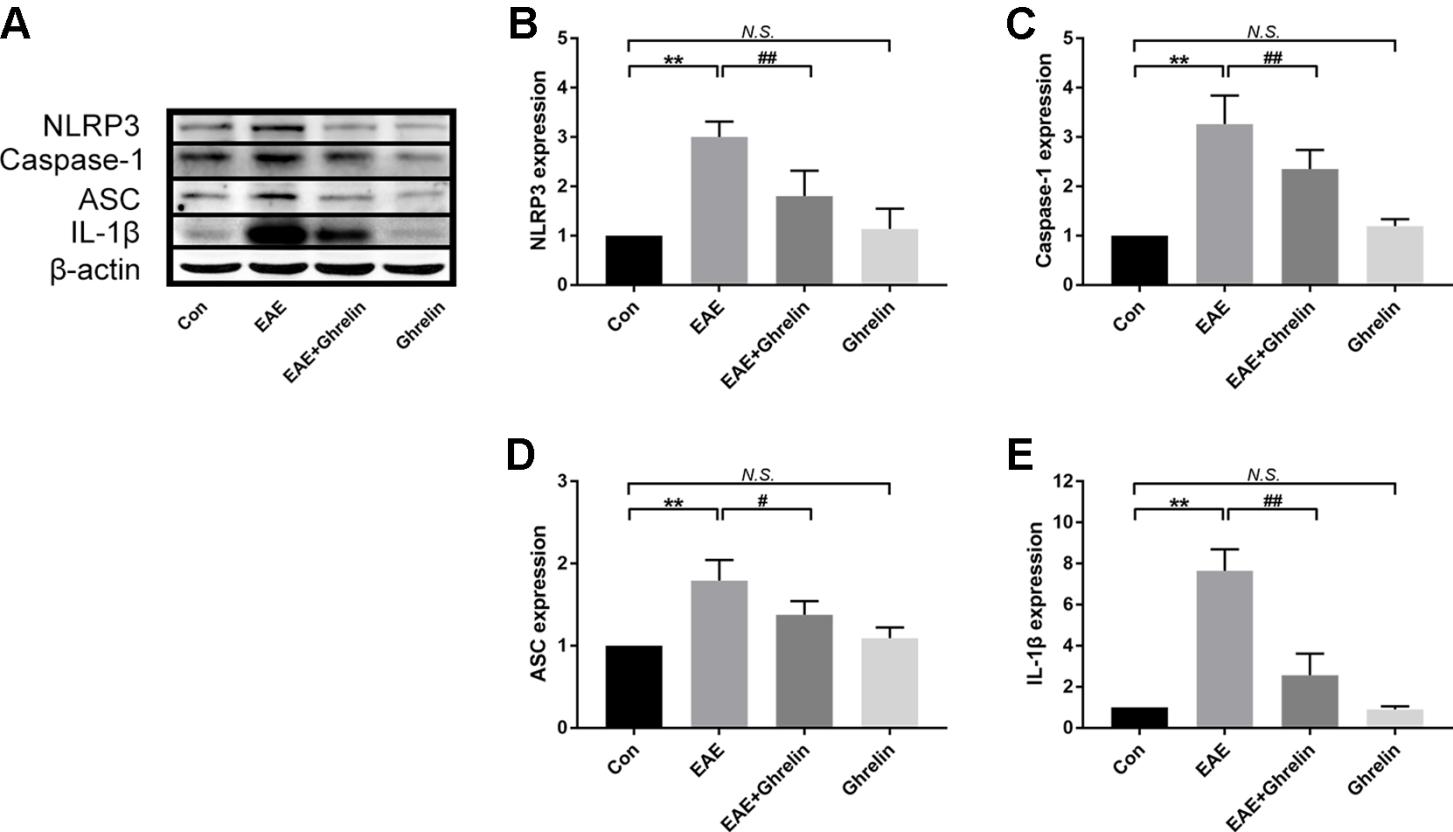
Treatment of ghrelin inhibited the NLRP3 signaling pathway in EAE rats. **(A)** Representative western blot images of changes in the expression level of NLRP3, ASC, caspase-1, and IL-1β. **(B–E)** β-Actin was used as internal control to calculate relative expression. Data were shown as mean ± SD (*n* = 5). **P 0.01 versus the control group, ^#^ *P* 0.05,^##^ *P* 0.01 versus the EAE group. *NS*, not significant. Con, control.

**Figure 5:**
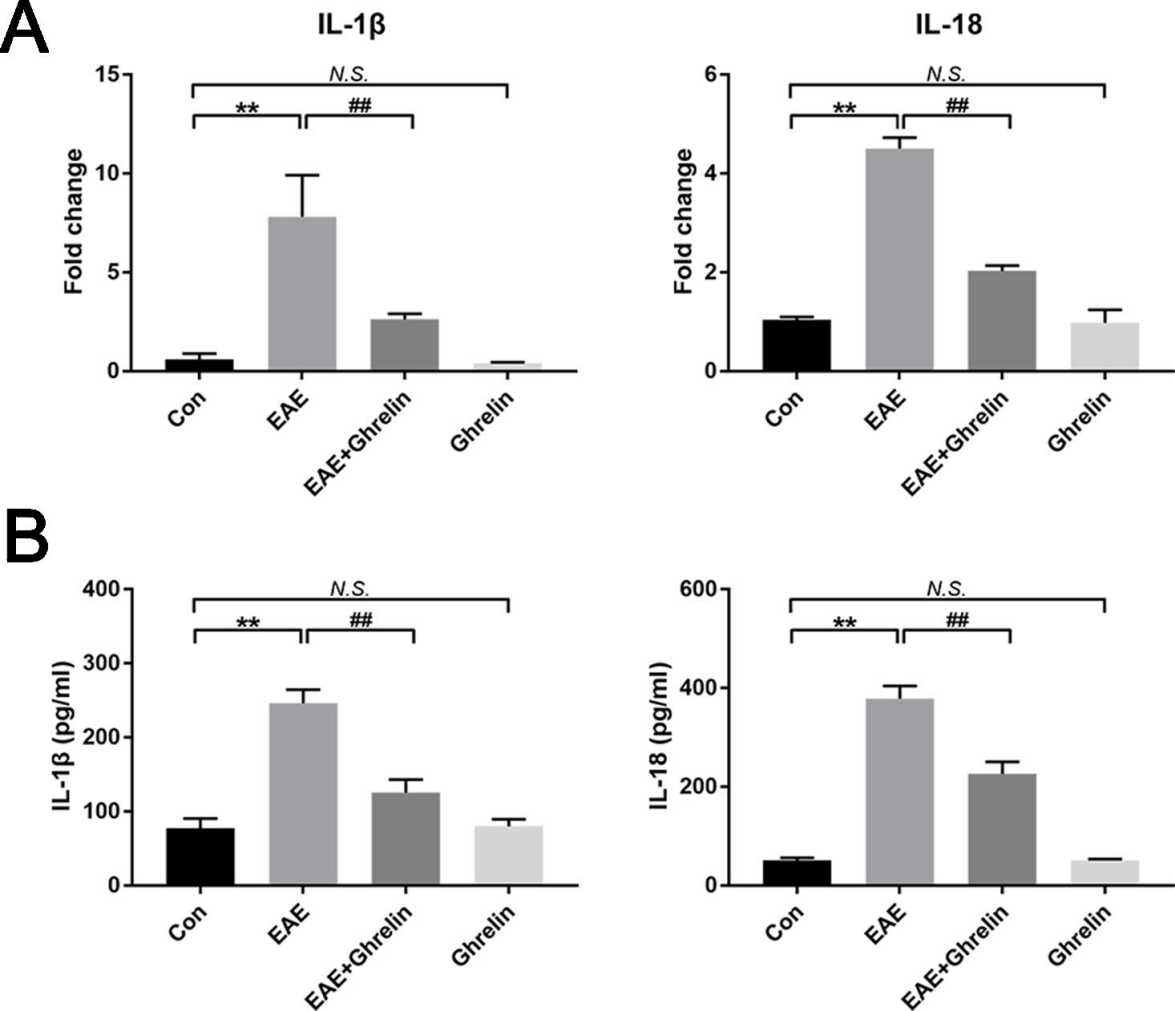
Treatment of ghrelin downregulated expressions of IL-1β and IL-18. **(A)** The mRNA expressions of IL-1β and IL-18 were measured by quantitative RT-PCR (*n* = 5). **(B)** IL-1β and IL-18 expressions in serum were measured by ELISA (*n* = 10). Data were shown as mean ± SD. **P 0.01 versus the control group, ^##^ *P* 0.01 versus the EAE group. *NS*, not significant. Con, control.

We investigated the underlying mechanisms behind the neuroprotective effects of ghrelin, NLRP3, IL-1β, ASC, and caspase-1 in CNS tissues by western blot analysis and found that molecular expressions were significantly elevated in the EAE rats compared with healthy control rats (**Figure 4**). Moreover, we also further analyzed expressions of two vital cytokines, IL-1β and IL-18, involved in the NLRP3 inflammasome signaling pathway. High IL-1β and IL-18 expression levels were noticed in the spinal cord tissues of EAE rats by RT-PCR (**Figure 5A**) and ELISA analysis (**Figure 5B**). Ghrelin inhibited increases in the expression of these cytokines, which provides a deeper understanding of ghrelin’s anti-inflammatory effects.

### Ghrelin Alleviates Pyroptosis in EAE Rats

**Figure 6:**
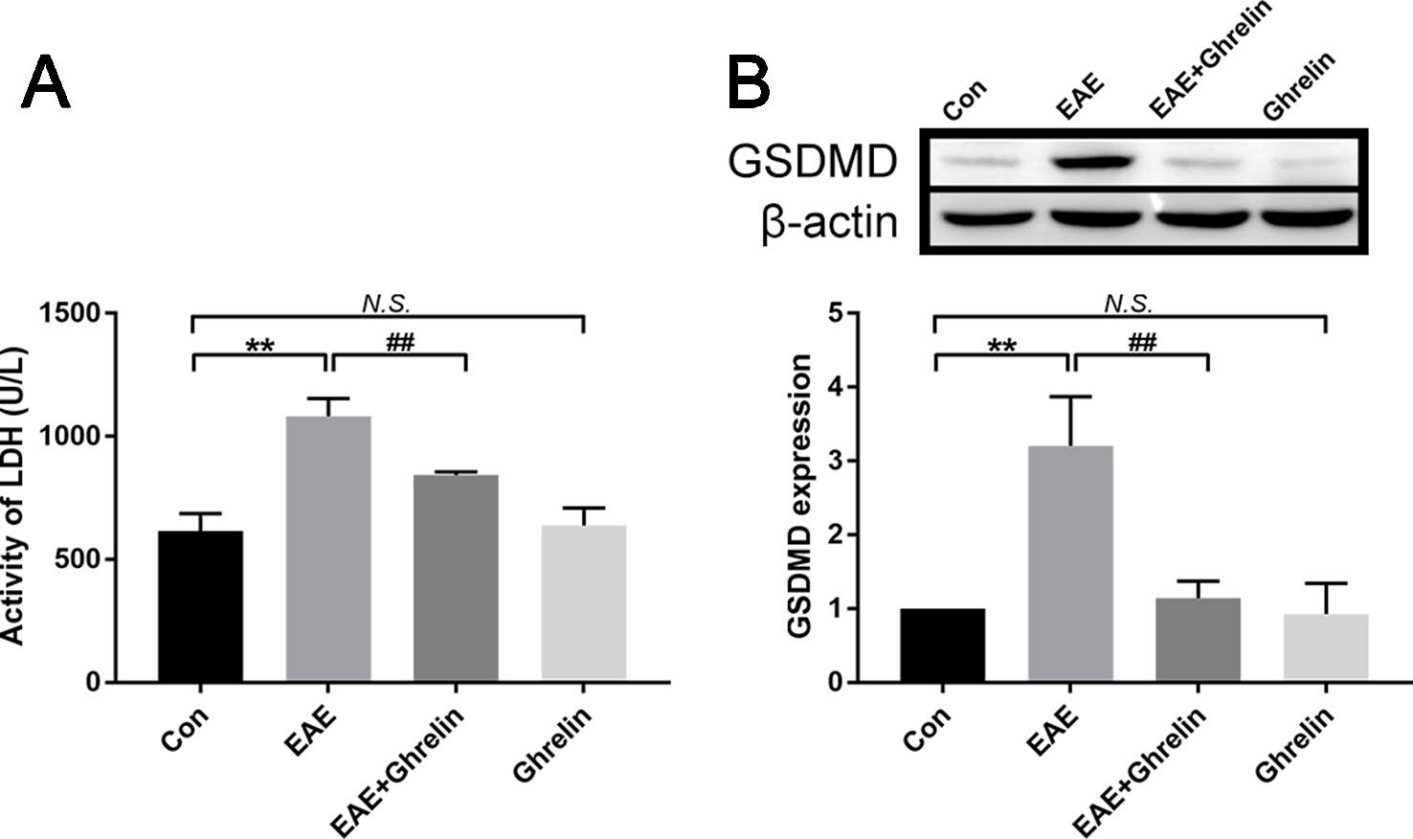
Treatment of ghrelin inhibited pyroptosis in EAE rats. **(A)** Expression of LDH in serum was measured by an LDH assay kit, *n* = 10. **(B)** Representative western blot image of changes in the expression level of GSDMD. β-Actin was used as internal control to calculate relative expression (*n* = 5). Data were shown as mean ± SD.**P 0.01 versus the control group, ^##^ *P* 0.01 versus the EAE group. *NS*, not significant. Con, control.

It is important to comprehend the relationship between ghrelin and pyroptosis during the onset period of EAE. We contrasted levels of LDH in serum and GSDMD in spinal cord tissue among these experimental groups. Compared with that in healthy control rats, expression of LDH in serum of EAE rats was relatively high. Treatment with ghrelin inhibited EAE-induced LDH production, which meant ghrelin lessened the occurrence of cell death (**Figure 6A**). In order to have a further understanding about whether pyroptosis happened in EAE rats and whether ghrelin could attenuate pyroptosis, western blotting was utilized to analyze protein expression levels of GSDMD. As shown in **Figure 6B**, we noticed that GSDMD was higher in EAE rats and lower in ghrelin-treated EAE rats. Therefore, we concluded that ghrelin could alleviate pyroptosis in EAE rats.

### Ghrelin Inhibits Activation of NF-κb in EAE Rats

**Figure 7:**
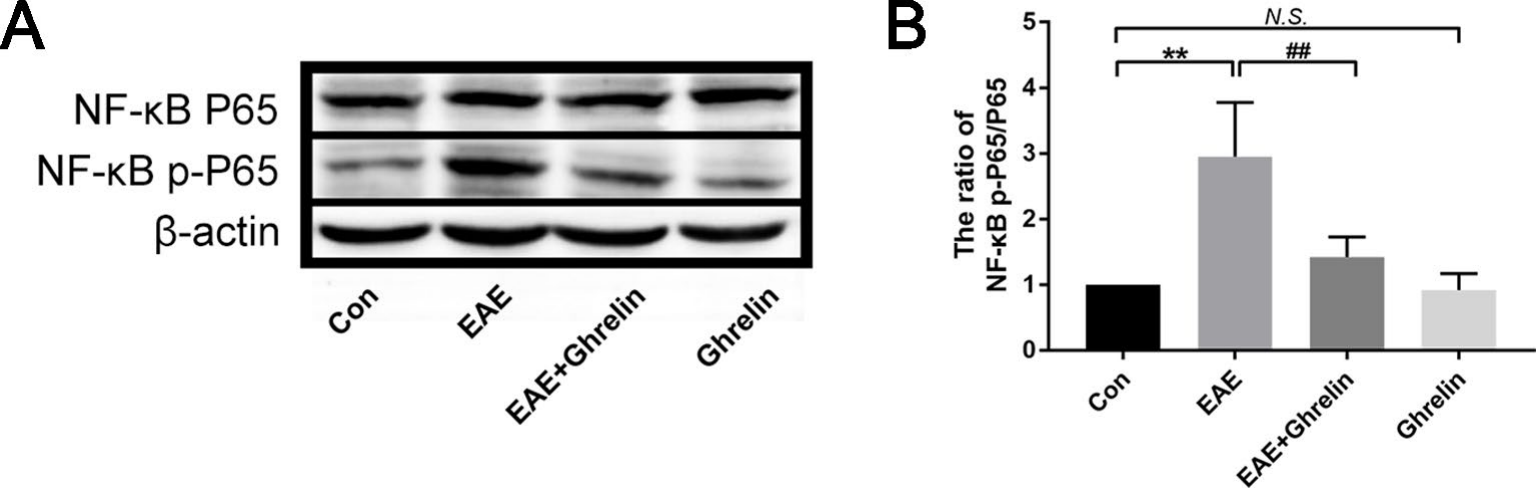
Treatment of ghrelin inhibited NF-κB activation in EAE rats. **(A)** Representative western blot images of changes in the expression level of NF-κBP65 and NF-κB p-P65. **(B)** The ratio of NF-κB p-P65/NF-κBP65. Data were shown as mean ± SD (*n* = 5).**P 0.01 versus the control group, ^##^ *P* 0.01 versus the EAE group. *NS*, not significant. Con, control.

The transcription factor NF-κB is related with many neuroinflammatory disorders and plays a key role in activating the NLRP3 inflammasome signaling pathway. In our study, we found that expression of NF-κB p-P65 in the spinal cord of EAE rats went up obviously (**Figures 7A, B**). Ghrelin administration reduced NF-κB p-P65 expression with statistical significance, which confirmed the neuroprotective effects of ghrelin against EAE.

### Ghrelin Has No Unconcerned Effects on Healthy Rats

Previous studies supported that ghrelin could increase appetite and weight (Klok et al., 2007; Howick et al., 2017). Because weight changes is an important evaluation indicator for disease severity, we administrated healthy rats with the same dose of ghrelin in order to eliminate bias that would influence the outcome. As shown in **Figures 1A, B**, difference of weight changes and neurological scores between healthy rats and the ghrelin-vehicle group did not achieve statistical significance (*P* > 0.05). In addition, results of histological examination (**Figures 1C–F**, *P* > 0.05), production of pro-inflammatory cytokines (**Figure 2**, *P* > 0.05), and molecules involved in the NLRP3 signaling pathway (**Figures 4**–**7**, *P* > 0.05) were not significant as well. These all revealed that the ghrelin dosage used in our experiments did not exert unconcerned effects on healthy rats.

## Discussion

In this study, we found that ghrelin administration markedly attenuated inflammation and demyelination in the CNS of EAE rats, as shown by the inhibition of inflammatory factors, microglial activation, and improved functional behaviors. Moreover, ghrelin influenced the NF-κB expression and thus suppressed the NLRP3 inflammasome signaling pathway and pyroptosis. These findings clarified the relationship between ghrelin and the NLRP3 inflammasome in MS, which serves as preclinical research to combat MS.

Neuroinflammation, one of the most basic protective responses of the CNS, plays a crucial role in many physiologic and pathologic processes (Niranjan, 2018). The brain and spinal cord can exert immune responses against injuries as a mechanism of self-defense, and these responses include the elimination of invasive pathogens, removal of damaged cells, and promotion of tissue repair. However, the neuroinflammatory process is a double-edged sword, which during unbalanced conditions, can result in varying degrees of tissue damage and dysfunction (DiSabato et al., 2016). MS is a neurodegenerative disease of the CNS that can cause permanent disabilities and primarily affects young people. With characteristic recurrence and progression, MS leaves patients with lifelong treatments and great suffering. An exaggerated neuroinflammatory response has been recognized as an etiologic factor of MS, and controlling inflammation is a primary therapeutic goal in the early stages of the disease. However, the therapeutic outcomes are not very satisfactory, and an urgent need for the research and development of new drugs exists.

Ghrelin, a novel gastrointestinal hormone, is the only endogenous ligand known for GHSR, to date. In addition to its endocrine actions, ghrelin plays multifactorial functions in different regions of the brain as a brain–gut peptide (Andrews, 2011). Positive functions of ghrelin involve anti-oxidation, anti-inflammation, anti-apoptosis, and autophagy enhancement effects (Jiao et al., 2017). In the CNS, ghrelin also plays these important roles, which has been verified experimentally in animal models of cerebral ischemia, traumatic brain injury, acute spinal cord injury, epilepsy, Parkinson’s disease, and Alzheimer’s disease (Moon et al., 2009; Lee et al., 2010; Portelli et al., 2012; Kenny et al., 2013; Cecarini et al., 2016; Shi et al., 2017b). However, there are only a limited number of studies looking at the effects of ghrelin against MS. The EAE animal, as a model for MS, has been widely adopted by researchers. In our study, spinal cords obtained from guinea pigs served as the major antigenic component to create the symptoms of EAE and then evaluate the neuroprotective efficacy of ghrelin.

After EAE rats were injected subcutaneously with ghrelin for several consecutive days, we observed improved body weight changes and neurologic scores compared with untreated ones. Moreover, brain inflammatory infiltrations, as well as the degree of demyelination in spinal cords, were also alleviated. As expected, the expressions of some pro-inflammatory mediators, including TNF-α, IL-6, and COX-2, were decreased in the spinal cord and serum of the EAE + ghrelin group rats compared with those of the EAE group rats, which further reinforced the anti-inflammatory actions of ghrelin. Under normal CNS conditions, NO regulates several physiologic processes, such as blood flow, immune responses, and synaptic transmissions. However, there is also growing evidence that high NO levels induced by iNOS are associated with many pathologic manifestations of MS (Smith and Lassmann, 2002). Similarly, our experiments verified that NO was much higher in EAE rats as detected by serum nitrite expression. According to its function of relieving EAE symptoms, ghrelin downregulated production of both NO and iNOS. Therefore, we concluded that the predominantly improved anti-inflammatory CNS capacity might be attributed to ghrelin neuroprotection. We also gave healthy rats an equivalent dose of ghrelin as that given to the EAE rats to assess whether ghrelin had any adverse or irrelevant effects that could influence the outcomes. We discovered that there were no side effects comparing with the healthy control group, as demonstrated by unnoticeable changes such as behavioral features, histopathological characteristics, and expressions of pro-inflammatory factors.

Pyroptosis is a newly discovered form of programmed cell death mediated by the pore-forming protein, GSDMD, which results in the production of numerous pro-inflammatory cytokines (Shi et al., 2015). It is divided into two types, the canonical pathway that depends on caspase-1 and the noncanonical pathway that depends on caspase-4, caspase-5, and caspase-11. Studies have shown that pyroptosis plays a key role in the occurrence and development of various diseases, such as infectious diseases, CNS diseases, and atherosclerosis. McKenzie et al. (2018) reported that pyroptosis in microglia and oligodendrocytes contributed to demyelination in EAE, and GSDMD inhibition could suppress pyroptosis in microglia. These findings fulfilled a new profile in the pathogenesis of MS. Inflammasome activation is increasingly thought to be the main step in initiating pyroptosis. The NLRP3 inflammasome is one of the most meticulously studied inflammasomes, and it contains three parts, namely, the NLRP3, ASC, and pro-caspase-1. Previous studies have revealed that microglia are closely related to the NLRP3 inflammasome. The combination of damage signals and pattern recognition receptors (PRRs) expressed on microglial cell membranes can activate the NLRP3 inflammasome. Therefore, the role of glial cells in neuroinflammation should not be neglected. Besides, numerous studies have reported that NLRP3 inflammasome was closely related with spinal cord demyelination. Enhanced and accelerated EAE development and spinal cord demyelination have been observed in NLRP3-deficient mice (Gris et al., 2010; Inoue et al., 2012). IL-1β and IL-18 are important factors in the NLRP3 inflammasome signaling pathway and are derived from their precursors, pro-IL-1β and pro-IL-18, respectively, which can be activated by functional caspase-1. IL-1β has neurotoxic effects in the EAE model as it increases the permeability of the BBB and accelerates leukocyte infiltration. In this study, we assumed that ghrelin could control inflammatory responses by inhibiting microglial activation, the NLRP3 inflammasome, and GSDMD-related pyroptosis. Results of IHC revealed that activated microglia aggregated around the damaged tissues and small vessels in EAE rats which signified microglia were involved in inflammatory damage. We also observed increased expression of NLRP3, caspase-1, and GSDMD in EAE rats, which was consistent with the findings in the study by McKenzie et al., while NLRP3, caspase-1, and GSDMD expressions were downregulated in the spinal cords of ghrelin-treated EAE rats. The results of RT-PCR and ELISA also showed decreased expression of IL-1β and IL-18. These results were in agreement with our hypothesis. In addition, a recent study reported the protective effects of ghrelin in heart ischemia/reperfusion injury by inhibition of NLRP3 inflammasome activation (Wang et al., 2017). Moreover, Ling et al. demonstrated that ghrelin prevented collagen fibril accumulation and apoptosis by weakening NLRP3 inflammasome activation in unilateral ureteral obstruction-induced renal injury (Ling et al., 2019). Therefore, our results, once again, have underscored the beneficial effects of ghrelin on fighting the NLRP3 inflammasome and pyroptosis.

The NLRP3 inflammasome can be affected by a wide variety of upstream elements, such as NF-κB (Afonina et al., 2017). PRRs combined with their corresponding ligands can act as a first signal to mediate the translocation of NF-κB into the nucleus and initiate NLRP3, pro-IL-1β, and pro-IL-18 transcription to upregulate PRR expression. It has been shown that the NLRP3 inflammasome signaling pathway could be inhibited by suppression of NF-κB signaling. In this study, we reached a similar conclusion that ghrelin was also able to attenuate NF-κB p65 expression in the spinal cord tissues of the EAE rats, which, in turn, modulated the NLRP3 inflammasome.

Our study has some limitations. First, we only showed that ghrelin inhibited the NLRP3 inflammasome signaling pathway, and therefore, we are not sure whether these neuroprotective effects involved other inflammasomes which have been reported to participate in the pathogenesis of MS (Freeman et al., 2017; Vidmar et al., 2019). Second, a concrete mechanism of how ghrelin interacts with neurons and glial cells to prevent EAE requires further exploration. Additionally, our study still has a timing limitation as the experimental observation was stopped when the neurological score was still increasing and not later on. More studies are warranted to assess whether ghrelin is really protecting animals or is just delaying disease onset, so that we can have a comprehensive understanding of its protective effectiveness.

**Figure 8:**
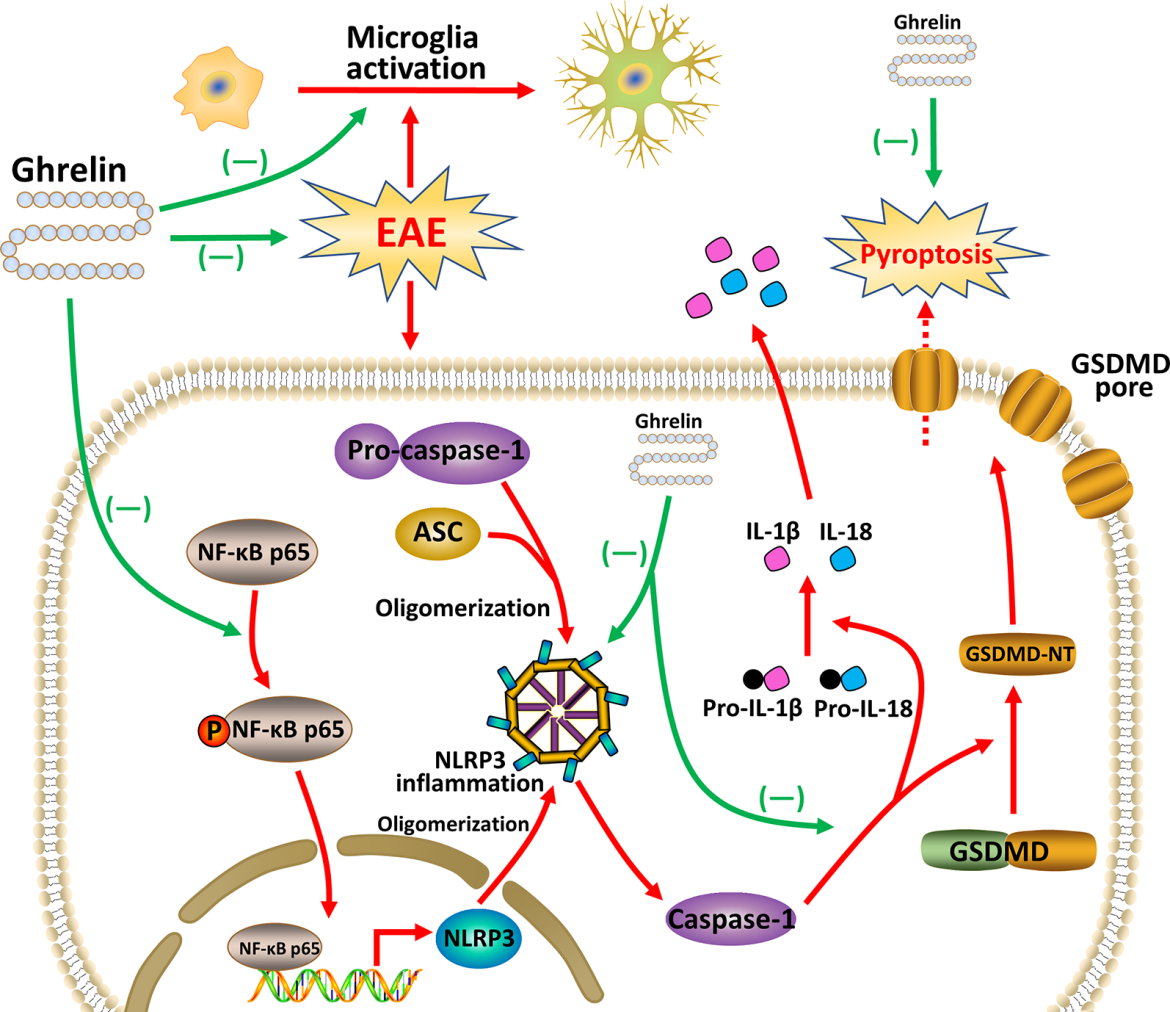
Ghrelin attenuates neuroinflammation and demyelination in EAE involving the NLRP3 inflammasome signaling pathway and pyroptosis.

In conclusion, our research concentrated on the therapeutic effects of ghrelin, including its anti-demyelination, anti-neuroinflammatory, and anti-pyroptosis effects. Combined results confirmed that ghrelin could suppress NF-κB, NLRP3 inflammasome signaling pathway, and pyroptosis, along with secretions of pro-inflammatory cytokines. Owing to the neuroprotective effects of ghrelin, EAE rats had delayed disease onsets and improved behavioral functions (**Figure 8**). Given the increasing evidence that ghrelin plays a significant role in complex CNS disorders, we provided additional insights into the functions of ghrelin as a novel therapeutic candidate for MS patients.

## Data Availability Statement

The datasets generated for this study are available on request to the corresponding author.

## Ethics Statement

The animal study was reviewed and approved by Institutional Animal Care and Use Committee of Shengjing Hospital, China Medical University.

## Author Contributions

FL and JF conceived and designed the entire study. FL, ZL, and HY collected and analyzed data. FL wrote the manuscript. XH and JF revised the original manuscript. All authors have read and approved the final submitted version.

## Funding

Our study was supported financially by the Program of Basic and Clinical Research Platform of China Medical University—the Second Batch of Basic Clinical Closely Combined Platform Project (CMU-201406), Animal Experimental Research and Clinical Application of Major Diseases (2012225021) and study on the epigenetic molecular mechanism of tanshinone IIA for the treatment of EAE, BBB destruction, and inflammatory immune responses funded by the Science and Technology Department of Liaoning Province (2018225002).

## Conflict of Interest

The authors declare that the research was conducted in the absence of any commercial or financial relationships that could be construed as a potential conflict of interest.

## References

[B1] Afonina I. S. Zhong Z. Karin M. Beyaert R. (2017). Limiting inflammation-the negative regulation of NF-kappaB and the NLRP3 inflammasome. Nat. Immunol. 18 (8), 861–869. 10.1038/ni.3772 28722711

[B2] Andrews Z. B. (2011). The extra-hypothalamic actions of ghrelin on neuronal function. Trends Neurosci. 34 (1), 31–40. 10.1016/j.tins.2010.10.001 21035199

[B3] Baker D. Amor S. (2014). Experimental autoimmune encephalomyelitis is a good model of multiple sclerosis if used wisely. Mult Scler Relat. Disord. 3 (5), 555–564. 10.1016/j.msard.2014.05.002 26265267

[B4] Barclay W. Shinohara M. L. (2017). Inflammasome activation in multiple sclerosis and experimental autoimmune encephalomyelitis (EAE). Brain Pathol. 27 (2), 213–219. 10.1111/bpa.12477 27997058 PMC8029098

[B5] Cecarini V. Bonfili L. Cuccioloni M. Keller J. N. Bruce-Keller A. J. Eleuteri A. M. (2016). Effects of ghrelin on the proteolytic pathways of Alzheimer’s disease neuronal cells. Mol. Neurobiol. 53 (5), 3168–3178. 10.1007/s12035-015-9227-x 26033219

[B6] Chang L. Niu F. Chen J. Cao X. Liu Z. Bao X. (2019). Ghrelin improves muscle function in dystrophin-deficient mdx mice by inhibiting NLRP3 inflammasome activation. Life Sci. 232, 116654. 10.1016/j.lfs.2019.116654 31306657

[B7] Chen X. He W. T. Hu L. Li J. Fang Y. Wang X. (2016). Pyroptosis is driven by non-selective gasdermin-D pore and its morphology is different from MLKL channel-mediated necroptosis. Cell Res. 26 (9), 1007–1020. 10.1038/cr.2016.100 27573174 PMC5034106

[B8] Correale J. Gaitan M. I. Ysrraelit M. C. Fiol M. P. (2017). Progressive multiple sclerosis: from pathogenic mechanisms to treatment. Brain 140 (3), 527–546. 10.1093/brain/aww258 27794524

[B9] DellaValle B. Brix G. S. Brock B. Gejl M. Landau A. M. Moller A. (2016). Glucagon-like peptide-1 analog, liraglutide, delays onset of experimental autoimmune encephalitis in lewis rats. Front. Pharmacol. 7, 433. 10.3389/fphar.2016.00433 27917122 PMC5114298

[B10] DiSabato D. J. Quan N. Godbout J. P. (2016). Neuroinflammation: the devil is in the details. J. Neurochem. 139 Suppl 2, 136–153. 10.1111/jnc.13607 26990767 PMC5025335

[B11] Freeman L. Guo H. David C. N. Brickey W. J. Jha S. Ting J. P. (2017). NLR members NLRC4 and NLRP3 mediate sterile inflammasome activation in microglia and astrocytes. J. Exp. Med. 214 (5), 1351–1370. 10.1084/jem.20150237 28404595 PMC5413320

[B12] Goldstein J. L. Zhao T. J. Li R. L. Sherbet D. P. Liang G. Brown M. S. (2011). Surviving starvation: essential role of the ghrelin-growth hormone axis. Cold Spring Harb Symp Quant Biol. 76, 121–127. 10.1101/sqb.2011.76.010447 21785007

[B13] Gris D. Ye Z. Iocca H. A. Wen H. Craven R. R. Gris P. (2010). NLRP3 plays a critical role in the development of experimental autoimmune encephalomyelitis by mediating Th1 and Th17 responses. J. Immunol. 185 (2), 974–981. 10.4049/jimmunol.0904145 20574004 PMC3593010

[B14] He W. T. Wan H. Hu L. Chen P. Wang X. Huang Z. (2015). Gasdermin D is an executor of pyroptosis and required for interleukin-1beta secretion. Cell Res. 25 (12), 1285–1298. 10.1038/cr.2015.139 26611636 PMC4670995

[B15] He X. Yuan W. Li Z. Feng J. (2017). An autophagic mechanism is involved in the 6-hydroxydopamine-induced neurotoxicity *in vivo* . Toxicol. Lett. 280, 29–40. 10.1016/j.toxlet.2017.08.006 28802652

[B16] Howick K. Griffin B. T. Cryan J. F. Schellekens H. (2017). From belly to brain: targeting the ghrelin receptor in appetite and food intake regulation. Int. J. Mol. Sci. 18 (2), E273. 10.3390/ijms18020273 28134808 PMC5343809

[B17] Inoue M. Shinohara M. L. (2013). NLRP3 inflammasome and MS/EAE. Autoimmune Dis. 2013, 859145. 10.1155/2013/859145 23365725 PMC3556409

[B18] Inoue M. Williams K. L. Gunn M. D. Shinohara M. L. (2012). NLRP3 inflammasome induces chemotactic immune cell migration to the CNS in experimental autoimmune encephalomyelitis. Proc. Natl. Acad. Sci. U.S.A. 109 (26), 10480–10485. 10.1073/pnas.1201836109 22699511 PMC3387125

[B19] Jiao Q. Du X. Li Y. Gong B. Shi L. Tang T. (2017). The neurological effects of ghrelin in brain diseases: beyond metabolic functions. Neurosci. Biobehav. Rev. 73, 98–111. 10.1016/j.neubiorev.2016.12.010 27993602

[B20] Kenny R. Cai G. Bayliss J. A. Clarke M. Choo Y. L. Miller A. A. (2013). Endogenous ghrelin’s role in hippocampal neuroprotection after global cerebral ischemia: does endogenous ghrelin protect against global stroke? Am. J. Physiol. Regul. Integr. Comp. Physiol. 304 (11), R980–R990. 10.1152/ajpregu.00594.2012 23576609

[B21] Klok M. D. Jakobsdottir S. Drent M. L. (2007). The role of leptin and ghrelin in the regulation of food intake and body weight in humans: a review. Obes Rev. 8 (1), 21–34. 10.1111/j.1467-789X.2006.00270.x 17212793

[B22] Kojima M. Hosoda H. Date Y. Nakazato M. Matsuo H. Kangawa K. (1999). Ghrelin is a growth-hormone-releasing acylated peptide from stomach. Nature 402 (6762), 656–660. 10.1038/45230 10604470

[B23] Lassmann H. (1999). The pathology of multiple sclerosis and its evolution. Philos. Trans. R Soc. Lond B Biol. Sci. 350 (1390), 1635–1640. 10.1098/rstb.1999.0508 PMC1692680 10603616

[B24] Lee J. Y. Chung H. Yoo Y. S. Oh Y. J. Oh T. H. Park S. (2010). Inhibition of apoptotic cell death by ghrelin improves functional recovery after spinal cord injury. Endocrinology 151 (8), 3815–3826. 10.1210/en.2009-1416 20444938

[B25] Li Z. Liu F. He X. Yang X. Shan F. Feng J. (2019). Exosomes derived from mesenchymal stem cells attenuate inflammation and demyelination of the central nervous system in EAE rats by regulating the polarization of microglia. Int. Immunopharmacol 67, 268–280. 10.1016/j.intimp.2018.12.001 30572251

[B26] Ling L. Yang M. Ding W. Gu Y. (2019). Ghrelin attenuates UUO-induced renal fibrosis *via* attenuation of Nlrp3 inflammasome and endoplasmic reticulum stress. Am. J. Transl. Res. 11 (1), 131–141. 30787974 PMC6357333

[B27] McKenzie B. A. Mamik M. K. Saito L. B. Boghozian R. Monaco M. C. Major E. O. (2018). Caspase-1 inhibition prevents glial inflammasome activation and pyroptosis in models of multiple sclerosis. Proc. Natl. Acad. Sci. U.S.A. 115 (26), E6065–E6074. 10.1073/pnas.1722041115 PMC6042136 29895691

[B28] Moon M. Kim H. G. Hwang L. Seo J. H. Kim S. Hwang S. (2009). Neuroprotective effect of ghrelin in the 1-methyl-4-phenyl-1,2,3,6-tetrahydropyridine mouse model of Parkinson’s disease by blocking microglial activation. Neurotox Res. 15 (4), 332–347. 10.1007/s12640-009-9037-x 19384567

[B29] Niranjan R. (2018). Recent advances in the mechanisms of neuroinflammation and their roles in neurodegeneration. Neurochem. Int. 120, 13–20. 10.1016/j.neuint.2018.07.003 30016687

[B30] Portelli J. Michotte Y. Smolders I. (2012). Ghrelin: an emerging new anticonvulsant neuropeptide. Epilepsia 53 (4), 585–595. 10.1111/j.1528-1167.2012.03423.x 22416903

[B31] Qiu X. Guo Q. Liu X. Luo H. Fan D. Deng Y. (2018). Pien Tze Huang alleviates relapsing-remitting experimental autoimmune encephalomyelitis mice by regulating Th1 and Th17 cells. Front. Pharmacol. 9, 1237. 10.3389/fphar.2018.01237 30429789 PMC6220046

[B32] Rhea E. M. Salameh T. S. Gray S. Niu J. Banks W. A. Tong J. (2018). Ghrelin transport across the blood-brain barrier can occur independently of the growth hormone secretagogue receptor. Mol. Metab. 18, 88–96. 10.1016/j.molmet.2018.09.007 30293893 PMC6308033

[B33] Scerif M. Goldstone A. P. Korbonits M. (2011). Ghrelin in obesity and endocrine diseases. Mol. Cell Endocrinol. 340 (1), 15–25. 10.1016/j.mce.2011.02.011 21345363

[B34] Semino C. Carta S. Gattorno M. Sitia R. Rubartelli A. (2018). Progressive waves of IL-1beta release by primary human monocytes *via* sequential activation of vesicular and gasdermin D-mediated secretory pathways. Cell Death Dis. 9 (11), 1088. 10.1038/s41419-018-1121-9 30352992 PMC6199333

[B35] Shi J. Gao W. Shao F. (2017a). Pyroptosis: gasdermin-mediated programmed necrotic cell death. Trends Biochem. Sci. 42 (4), 245–254. 10.1016/j.tibs.2016.10.004 27932073

[B36] Shi J. Zhao Y. Wang K. Shi X. Wang Y. Huang H. (2015). Cleavage of GSDMD by inflammatory caspases determines pyroptotic cell death. Nature 526 (7575), 660–665. 10.1038/nature15514 26375003

[B37] Shi L. Du X. Jiang H. Xie J. (2017b). Ghrelin and neurodegenerative disorders-a review. Mol. Neurobiol. 54 (2), 1144–1155. 10.1007/s12035-016-9729-1 26809582

[B38] Smith K. J. Lassmann H. (2002). The role of nitric oxide in multiple sclerosis. Lancet Neurol. 1 (4), 232–241. 10.1016/s1474-4422(02)00102-3 12849456

[B39] Song L. Pei L. Yao S. Wu Y. Shang Y. (2017). NLRP3 Inflammasome in neurological diseases, from functions to therapies. Front. Cell. Neurosci. 11, 63. 10.3389/fncel.2017.00063 28337127 PMC5343070

[B40] Souza-Moreira L. Delgado-Maroto V. Morell M. O’Valle F. Del Moral R. G. Gonzalez-Rey E. (2013). Therapeutic effect of ghrelin in experimental autoimmune encephalomyelitis by inhibiting antigen-specific Th1/Th17 responses and inducing regulatory T cells. Brain Behav. Immun. 30, 54–60. 10.1016/j.bbi.2013.01.080 23376169

[B41] Steyn F. J. Tolle V. Chen C. Epelbaum J. (2016). Neuroendocrine regulation of growth hormone secretion. Compr Physiol. 6 (2), 687–735. 10.1002/cphy.c150002 27065166

[B42] Vidmar L. Maver A. Drulovic J. Sepcic J. Novakovic I. Ristic S. (2019). Multiple sclerosis patients carry an increased burden of exceedingly rare genetic variants in the inflammasome regulatory genes. Sci. Rep. 9 (1), 9171. 10.1038/s41598-019-45598-x 31235738 PMC6591387

[B43] Wang Q. Lin P. Li P. Feng L. Ren Q. Xie X. (2017). Ghrelin protects the heart against ischemia/reperfusion injury *via* inhibition of TLR4/NLRP3 inflammasome pathway. Life Sci. 186, 50–58. 10.1016/j.lfs.2017.08.004 28782532

[B44] Wang S. Yuan Y. H. Chen N. H. Wang H. B. (2019). The mechanisms of NLRP3 inflammasome/pyroptosis activation and their role in Parkinson’s disease. Int. Immunopharmacol 67, 458–464. 10.1016/j.intimp.2018.12.019 30594776

[B45] Yang X. Yan J. Feng J. (2016). Treatment with tanshinone IIA suppresses disruption of the blood-brain barrier and reduces expression of adhesion molecules and chemokines in experimental autoimmune encephalomyelitis. Eur. J. Pharmacol. 771, 18–28. 10.1016/j.ejphar.2015.12.014 26683637

